# Efficient and Highly Specific Gene Transfer Using Mutated Lentiviral Vectors Redirected with Bispecific Antibodies

**DOI:** 10.1128/mBio.02990-19

**Published:** 2020-01-21

**Authors:** Christina L. Parker, Timothy M. Jacobs, Justin T. Huckaby, Dimple Harit, Samuel K. Lai

**Affiliations:** a Division of Pharmacoengineering and Molecular Pharmaceutics, Eshelman School of Pharmacy, University of North Carolina at Chapel Hill, Chapel Hill, North Carolina, USA; b Department of Biomedical Engineering, University of North Carolina at Chapel Hill, Chapel Hill, North Carolina, USA; c Department of Microbiology and Immunology, University of North Carolina at Chapel Hill, Chapel Hill, North Carolina, USA; University of Delaware

**Keywords:** lentivirus, bispecific antibody, gene therapy, Sindbis, targeted gene delivery

## Abstract

Despite their exceptional potencies, the broad tropism of most commonly used lentivirus (LV) vectors limits their use for targeted gene delivery *in vivo*. We hypothesized that we could improve the specificity of LV targeting by coupling (i) reduction of their binding to off-target cells with (ii) redirection of the vectors with a bispecific antibody (bsAb) that binds both LV and receptors on target cells. As a proof of concept, we pseudotyped nonreplicating LV using a mutated Sindbis envelope (mSindbis) with ablated binding to native receptors, while retaining the capacity to facilitate efficient fusion and endosomal escape. We then evaluated the transduction potencies of the mSindbis LV for HER2-positive (HER2^+^) (SKBR3) breast and HER2-negative (HER2^−^) (A2780) cells when redirected with different bsAbs. mSindbis LV alone failed to induce appreciable green fluorescent protein (GFP) expression in either cell. When mixed with HER2-targeting bsAb, mSindbis LV was exceptionally potent, transducing 12% to 16% of the SKBR3 cells at a multiplicity of infection (MOI [ratio of viral genome copies to target cells]) of 3. Transduction was highly specific, resulting in ∼50-fold-greater selectivity toward SKBR3 cells versus A2780 cells. Redirecting mSindbis LV led to a 10-fold improvement in cell-specific targeting compared to redirecting wild-type Sindbis LV with the same bsAb, underscoring the importance of ablating native virus tropism in order to maximize targeting specificity. The redirection of mutated LV using bsAb represents a potent and highly versatile platform for targeted gene therapy.

## Abstract (precis)

The goal of gene therapy is specific delivery and expression of therapeutic genes to target cells and tissues. Common lentivirus (LV) vectors are efficient gene delivery vehicles but offer little specificity. Here, we report an effective and versatile strategy to redirect LV to target cells using bispecific antibodies (bsAbs) that bind both cell receptors and LV envelope domains. Importantly, we ablated the native receptor binding of LV to minimize off-target transduction. Coupling bsAb specificity and ablated native LV tropism synergistically enhanced the selectivity of our targeted gene delivery system. The modular nature of our bsAb-based redirection enables facile targeting of the same LV to diverse tissues/cells. By abrogating the native broad tropism of LV, our bsAb-LV redirection strategy may enable lentivirus-based gene delivery *in vivo*, expanding the current use of LV beyond *ex vivo* applications.

## INTRODUCTION

Selective transduction of only target cells and tissues represents a major goal of therapeutic gene delivery. To achieve selective transduction, gene vectors must avoid binding to off-target cells while quickly binding to target cells with high specificity and must efficiently deliver DNA to the nucleus following cell entry. Among common viral vectors, lentivirus (LV) represents one of the most efficient gene transduction systems for stable, long-term transgene expression (1–3). Importantly, the safety of LV use has greatly improved (1, 2, 4) since adverse effects were first observed in patients with X-linked clinical severe combined immunodeficiency (SCID) who underwent retrovirus-mediated gene therapy (5–11). As a result, LV vectors are now routinely used in chimeric antigen receptor (CAR) T-cell therapies for treatment of B-cell malignancies where cells are selected, transduced with LV vectors, expanded, and reinfused into patients; two such therapies have already received regulatory approval.

Despite the routine *in vivo* delivery of cells transduced with LV vectors *ex vivo*, LV vectors are rarely used directly for *in vivo* gene therapy. This is because common LV vectors lack cell specificity: wild-type (WT) LV envelope proteins generally bind proteins ubiquitously present on the surface of most cells, leading to extensive off-target effects. Strategies to alter or restrict the natural tropism of LV vectors include either pseudotyping LV with different viral envelope proteins possessing altered tropism and biodistribution (3, 12) or genetically inserting ligands, peptides, and single-chain antibodies (Abs) into viral envelope glycoprotein domains to confer new cellular specificity (13–19). Unfortunately, introducing large proteins can be deleterious to the structure of viral proteins, can impede proper folding of the incorporated peptide that diminishes cell binding, and may hinder viral infectivity by altering normal functions of viral attachment proteins or preventing conformational changes necessary for fusion (3). Indeed, modified vectors can suffer from inconsistent specificity, reduced fusion activity, and low viral titers (17, 20). Not surprisingly, the success of modifying viral envelope glycoprotein domains critically depends on the size, structure, and binding activity of ligand.

To enable highly specific transduction, we believe that we must confer cell-specific receptor binding while simultaneously minimizing off-target binding. With wild-type viral vectors that are either pseudotyped with Ab or mixed with adaptor molecules, the resulting vectors can still bind and transduce off-target cells/tissues via the native viral Env. Thus, to further improve viral vector specificity, it is important to first minimize nonspecific binding of LV to off-target cells. Previous work has shown that mutations in the receptor-binding domain (E2) of the Sindbis glycoprotein structure (mSindbis) eliminated its natural tropism for the liver and spleen without affecting viral assembly or its high level of titer production (13, 21). These mutations specifically targeted regions within the E2 domain known to mediate binding to target cells and are sites for neutralizing antibodies (13).

To create a versatile redirection platform, we combined mSindbis-pseudotyped LV with bispecific antibodies (bsAbs) that bind both mSindbis E2 and specific cell receptors. E2- and HER2-specific bsAbs provide the specificity required to redirect mSindbis LV to transduce HER2-positive (HER2^+^) cells, thus enabling the use of LV with an unmodified viral envelope that likely maximizes stability, high titer production, and efficient transduction. A longstanding challenge in bsAb engineering has been the proper pairing of heavy and light chains to lead to high purity and yield of the final product. We used a recently developed bsAb platform called OrthoMab to investigate targeted viral gene delivery to select cells using bispecific antibodies. By introducing orthogonal mutation pairs into heavy and light chains, the OrthoMab platform yields high-fidelity pairing of the correct heavy and light chains for functional bsAbs. Here, we report a versatile gene carrier system, based on combining bsAb with LV, that facilitates highly potent and specific gene delivery.

## RESULTS

### OrthoMab-based bsAbs preserve specificity and affinity to antigens.

**FIG 1:**
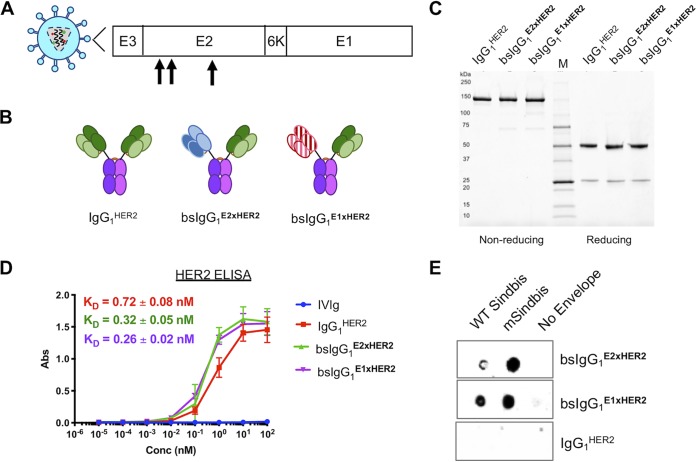
Characterization of control antibodies (Abs) and bispecific antibodies (bsAbs). (A) Schematic representation of Sindbis glycoprotein domains E1 and E2. Mutated Sindbis envelope glycoprotein (mSindbis) contains mutations in the E2 domain (indicated by arrows) that ablate native receptor binding. The E1 domain forms a heterodimer with E2, and E3 is a signal sequence peptide for E2 protein. (B) Schematic of control and bispecific Ab illustrating size and key design features. (C) Nonreducing (left) and reducing (right) protein gels showing Coomassie blue staining of control and bispecific Abs. (D) Binding affinity of control and bispecific Ab to HER2-Fc chimera analyzed by ELISA (*n* = 2). Conc, concentration. (E) Selective binding of αE2 and αE1 bispecific Ab to Sindbis pseudotyped lentiviruses and absence of binding to the negative control (no-envelope lentivirus) as visualized by dot blotting.

We first engineered chimeric bsAb against both (i) HER2 overexpressed on breast cancer cells and (ii) Sindbis Env glycoproteins displayed on LV. This was accomplished by merging human IgG_1_ backbones with HER2-negative (HER2^−^) and Sindbis envelope-binding V_H_ and V_L_ domains previously isolated from mouse IgG (22). We prepared bsAb that bound either Sindbis Env glycoprotein E1 domain (responsible for pH-dependent endolysosomal membrane fusion and escape) or E2 domain (responsible for binding high-affinity laminin receptors [23] or heparin sulfate [24] for cellular entry) (Fig. 1A). Purified bsAbs were separated via size exclusion chromatography and exhibited the expected molecular sizes as visualized on nonreduced and reduced protein gels (Fig. 1B and C).

We next confirmed the specificity and affinity of the bsAbs using antigen-specific enzyme-linked immunosorbent assays (ELISAs) against HER2. We found that both bispecific bsIgG_1_^E2xHER2^ and bsIgG_1_^E1xHER2^ possessed binding affinities to HER2 similar to those seen with the monoclonal anti-HER2 IgG_1_ (trastuzumab; IgG_1_^HER2^ control): the equilibrium dissociation constant (*K_D_*) values for bsIgG_1_^E2xHER2^, bsIgG_1_^E1xHER2^, and IgG_1_^HER2^ were 0.32 ± 0.05 nM, 0.26 ± 0.02 nM, and 0.72 ± 0.08 nM, respectively (Fig. 1D). We also assessed the binding of our bsAbs to WT Sindbis and mSindbis pseudotyped LV using dot blotting. Both bsAbs bound WT Sindbis and mSindbis Env pseudotyped LV and did not bind to LV without an envelope (i.e., negative control) (Fig. 1E). As expected, IgG_1_^HER2^ did not bind to WT Sindbis, mSindbis, or the nonenveloped LV control. Altogether, these results confirmed that we had prepared functional bsAbs and that the orthogonal mutations introduced at the interface of the heavy and light chains for both Fabs did not impair binding to either HER2 or Sindbis envelope.

### bsIgG_1_^E2xHER2^ enhanced mutated LV infectivity compared to wild-type LV alone.

**FIG 2:**
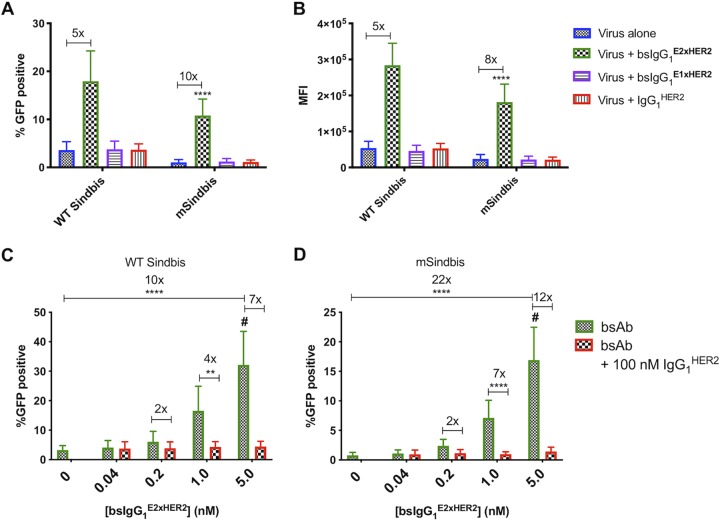
BsIgG_1_^E2xHER2^ enhanced transduction by WT Sindbis and mSindbis pseudotyped lentiviral vectors against HER2^+^ SKBR3 cells compared to either virus alone. (A and B) bsAb-mediated viral infectivity was measured by flow cytometry as (A) percentage of GFP-positive cells and (B) mean fluorescence intensity (MFI). Data represent results of *n* = 5 independent experiments performed in duplicate, MOI = 3, and antibody concentration = 1 nM (two-way ANOVA *post hoc* Tukey’s test. ****, *P* < 0.0001 [versus all conditions]). For statistical analysis of group comparisons across all treatment groups, see Table S1 in the supplemental material (see also Fig. 2A) and Table S2 (see also Fig. 2B). (C and D) Targeted lentiviral infectivity is dependent upon HER2 specificity of bsAb for WT Sindbis (C) and mSindbis (D). At all tested concentrations of bsAb, excess trastuzumab (αHER2 IgG_1_) effectively blocked viral infectivity of both targeted lentiviruses, suggesting that the infectivity was mediated specifically via binding to HER2 receptor and was not due to differences between lentiviruses. Data represent results of *n* = 3 independent experiments performed in duplicate (MOI = 3) and analyzed using two-way ANOVA with a *post hoc* Tukey’s test (#, *P* < 0.0001 [versus all conditions]; ****, *P* < 0.0001; **, *P* = 0.0013).

Using flow cytometry, we first measured the transduction efficiency of native, nontargeted WT Sindbis and mSindbis lentiviruses expressing green fluorescent protein (GFP) in HER2^+^ SKBR3 cells using a low vector-to-cell ratio (commonly referred to as multiplicity of infection [MOI]) of 3. As expected, mSindbis had markedly lower transduction efficiency than WT Sindbis, transducing only ∼1% of target HER2^+^ cells versus ∼4% for WT Sindbis, with 2-fold-lower mean fluorescence intensity (MFI) than WT Sindbis (Fig. 2A and B). The infectivity of both WT Sindbis LV and mSindbis LV was substantially enhanced when premixed with 1 nM E2-binding bsIgG_1_^E2xHER2^, resulting in transduction of ∼18% and ∼12% of HER2^+^ cells at the same MOI, respectively (Fig. 2A and B). Compared to nontargeted WT Sindbis, the redirected WT Sindbis transduced 5-fold more target cells, with 5-fold-greater MFI, whereas the redirected mSindbis transduced 10-fold more target cells than mSindbis alone, with 8-fold-greater MFI. These results indicate that bsAb can indeed confer greater cell binding of LV, with a more pronounced improvement seen for mSindbis than for WT Sindbis, most likely due to the exceedingly limited transduction by mSindbis LV alone. Targeted LV treatment also maintained a similar level of cytotoxicity compared to both untreated cells and cells treated with LV alone, suggesting that lentiviral redirection using bsAb is not toxic to cells (see Fig. S1 in the supplemental material).

10.1128/mBio.02990-19.1 FIG S1 Lentiviral redirection with bispecific antibodies exhibited minimal to no effect on cell viability compared to untreated cells. Immediately following a viral infectivity assay performed with bispecific antibodies in SKBR3 cells, the viability of untreated and transduced cells was measured using 3-(4,5-dimethylthiazolyl-2)-2,5-diphenyltetrazolium bromide (MTT) assay. Cells were incubated with 0.5 mg/ml MTT solution for 1 h at 37°C prior to the addition of isopropanol to dissolve formazan crystals, and absorbance was measured at 560 nm (signal) and 670 nm (background). Cell viability was reported as percent viability of treated cells relative to untreated cells. Data represent results of *n* = 2 independent experiments performed in triplicate. MOI = 3. Antibody concentration = 5 nM. For statistical analyses of group comparisons across all treatment groups, see Table S9. Download FIG S1, TIF file, 1.7 MB. Copyright © 2020 Parker et al. 2020 Parker et al. This is an open-access article distributed under the terms of the Creative Commons Attribution 4.0 International license.

10.1128/mBio.02990-19.2 TABLE S1 Statistical comparison analysis results for Fig. 2A. Group comparisons were analyzed using two-way ANOVA and *post hoc* Tukey’s test. Download Table S1, DOCX file, 0.01 MB. Copyright © 2020 Parker et al. 2020 Parker et al. This is an open-access article distributed under the terms of the Creative Commons Attribution 4.0 International license.

10.1128/mBio.02990-19.3 TABLE S2 Statistical comparison analysis results for Fig. 2B. Group comparisons were analyzed using two-way ANOVA and *post hoc* Tukey’s test. Download Table S2, DOCX file, 0.01 MB. Copyright © 2020 Parker et al. 2020 Parker et al. This is an open-access article distributed under the terms of the Creative Commons Attribution 4.0 International license.

We next assessed whether increasing the concentration of bsIgG_1_^E2xHER2^ could further enhance the transduction efficiency of both redirected LVs. At the highest bsIgG_1_^E2xHER2^ concentration tested, redirected WT Sindbis and mSindbis LV transduced ∼32% and ∼17% of SKBR3 cells, increasing the fraction of GFP^+^ SKBR3 cells by ∼10-fold and ∼22-fold, respectively, compared to the corresponding nontargeted LVs (Fig. 2C and D). BsIgG_1_^E2xHER2^ redirection was highly specific to HER2, as incubation with excess IgG_1_^HER2^ control effectively blocked transduction, reducing the percentage of GFP^+^ cells at each tested bsAb concentration to the level seen with nontargeted LVs (Fig. 2C and D).

To assess whether bsIgG simply needs to engage the LV or if efficient transduction is dependent on bsIgG binding to specific viral epitopes, we evaluated in parallel the transduction potencies of LVs premixed with bsIgG_1_^E1xHER2^. Interestingly, bsIgG_1_^E1xHER2^ did not improve the transduction efficiency of either LV at all, with percentages of GFP^+^ cells and MFI of transduced cells comparable to those seen with nontargeted LV alone (Fig. 2A and B). Nontargeted WT Sindbis, WT Sindbis mixed with bsIgG_1_^E1xHER2^, and WT Sindbis mixed with IgG_1_^HER2^ control all transduced ∼4% of HER2^+^ cells. Similarly, nontargeted mSindbis, mSindbis mixed with bsIgG_1_^E1xHER2^, and mSindbis mixed with IgG_1_^HER2^ control all transduced ∼1% of HER2^+^ cells. These results indicate that bsAb-mediated gene transfer by LV is critically dependent on bsAb engaging specific epitopes on the Sindbis Env-binding domain on the LV surface.

### Targeted LV vectors preferentially transduced target HER2^+^ cells.

**FIG 3:**
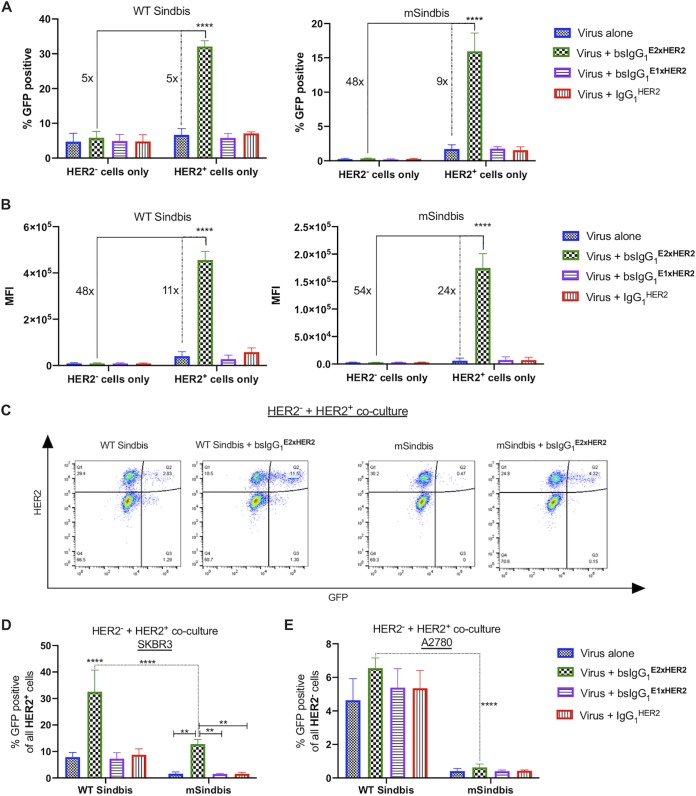
Specific infection of HER2^+^ cells in a mixed cell population. (A and B) Targeted WT Sindbis (A) and mSindbis (B) substantially enhanced viral infectivity in HER2^+^ cells compared to control HER2^−^ cells. Solid lines compare the selectivity of redirected LV in HER2^+^ cells to that in HER2^−^ cells, and dashed lines compare the transduction efficiency of redirected LV using bsIgG_1_^E2xHER2^ versus LV alone in target HER2^+^ cells. Viral infectivity was measured by flow cytometry as (A) percentage of GFP-positive cells and (B) mean fluorescence intensity (MFI). (C) A2780 (HER2^−^) cells were mixed with SKBR3 (HER2^+^) cells to create a mixed cell population. (D and E) Both targeted lentiviruses demonstrated selectivity for (D) HER2^+^ cells compared to (E) HER2^−^ cells as indicated by the substantial increase in the percentage of GFP-positive cells. Data represent results of 2 independent experiments performed in duplicate (MOI 3; [Ab] = 1 nM) and analyzed using two-way ANOVA with a *post hoc* Tukey’s test (****, *P* < 0.0001 [versus all conditions]; **, *P* = 0.0012). For statistical analysis of group comparisons across all treatment groups, see Table S3 (A), Table S4 (B), Table S5 (D), and Table S6 (E).

To evaluate the specificity of bsAb-mediated LV for target cells relative to off-target cells, we compared their transduction potencies on HER2^+^ (SKBR3) and HER2^−^ (A2780) cells separately, where A2780 represented a nonspecific cell control with little to no HER2 expression. As expected, we observed levels of transduction of HER2^−^ cells with WT Sindbis and mSindbis LV alone (5% and 0.2% of A2780 cells, respectively) that were comparable to those seen with HER2^+^ cells (7% and 1.7% of SKBR3 cells, respectively) (Fig. 3A). Premixing LV with bsIgG_1_^E2xHER2^ did not appreciably increase transduction of HER2^−^ cells, with 6% and 0.3% of A2780 cells transduced with redirected WT Sindbis and mSindbis LV (Fig. 3A). Compared to WT Sindbis LV alone, bsIgG_1_^E2xHER2^-targeted WT Sindbis increased the percentage of GFP^+^ cells by 5-fold (Fig. 3A, dotted line) and MFI by 11-fold (Fig. 3B, dotted line). Redirecting mSindbis LV with bsIgG_1_^E2xHER2^ led to a greater improvement over the results seen with mSindbis LV alone, with a 9-fold increase in the percentage of GFP^+^ cells (Fig. 3A, dotted line) and 24-fold higher MFI (Fig. 3B, dotted line). Both redirected LVs demonstrated markedly greater selectivity for HER2^+^ cells than for HER2^−^ cells, with the redirected mSindbis LV substantially exceeding the specificity of the targeted WT Sindbis LV. In particular, WT Sindbis LV redirected with bsIgG_1_^E2xHER2^ increased the percentage of GFP^+^ cells by 5-fold (Fig. 3A, solid line) and MFI by 48-fold (Fig. 3B, solid line) in HER2^+^ SKBR3 cells compared to HER2^−^ A2780 cells. Similarly, redirected mSindbis LV transduced 48-fold more SKBR3 cells than A2780 cells, with 54-fold higher MFI than mSindbis LV alone (Fig. 3A and B, solid lines).

10.1128/mBio.02990-19.4 TABLE S3 Statistical comparison analysis results for Fig. 3A. Group comparisons were analyzed using two-way ANOVA and *post hoc* Tukey’s test. Download Table S3, DOCX file, 0.01 MB. Copyright © 2020 Parker et al. 2020 Parker et al. This is an open-access article distributed under the terms of the Creative Commons Attribution 4.0 International license.

10.1128/mBio.02990-19.5 TABLE S4 Statistical comparison analysis results for Fig. 3B. Group comparisons were analyzed using two-way ANOVA and *post hoc* Tukey’s test. Download Table S4, DOCX file, 0.01 MB. Copyright © 2020 Parker et al. 2020 Parker et al. This is an open-access article distributed under the terms of the Creative Commons Attribution 4.0 International license.

10.1128/mBio.02990-19.6 TABLE S5 Statistical comparison analysis results for Fig. 3D. Group comparisons were analyzed using two-way ANOVA and *post hoc* Tukey’s test. Download Table S5, DOCX file, 0.01 MB. Copyright © 2020 Parker et al. 2020 Parker et al. This is an open-access article distributed under the terms of the Creative Commons Attribution 4.0 International license.

10.1128/mBio.02990-19.7 TABLE S6 Statistical comparison analysis results for Fig. 3E. Group comparisons were analyzed using two-way ANOVA and *post hoc* Tukey’s test. Download Table S6, DOCX file, 0.01 MB. Copyright © 2020 Parker et al. 2020 Parker et al. This is an open-access article distributed under the terms of the Creative Commons Attribution 4.0 International license.

To further assess the specificity of gene transfer, we evaluated bsIgG_1_^E2xHER2^-targeted LV to selectively transduce HER2^+^ cells in cocultures of both HER2^+^ and HER2^−^ cells. In good agreement with its broad transduction nature and with results from monoculture experiments, nontargeted WT Sindbis had very poor selectivity, transducing ∼8% of HER2^+^ cells (Fig. 3D) and ∼5% of HER2^−^ cells in this coculture setting (Fig. 3E). Nontargeted mSindbis LV also had relatively limited selectivity, transducing ∼2% of HER2^+^ cells (Fig. 3D) and ∼0.4% of HER2^−^ cells (Fig. 3E). Redirecting WT Sindbis LV with bsIgG_1_^E2xHER2^ modestly increased both the potencies and the specificity: targeted WT Sindbis LV exhibited ∼5× selectivity toward HER2^+^ cells, transducing ∼33% of SKBR3 cells versus ∼7% of A2780 cells (Fig. 3D and E). In contrast, combining bsAb-based redirection with ablation of native receptor binding synergistically enhanced targeting efficiencies, with ∼20× selectivity toward HER2^+^ in comparison to HER2^−^ cells (∼13% of SKBR3 cells versus ∼0.6% of A2780 cells) in this coculture study. Overall, redirected mSindbis LV was ∼2-fold more efficient in transducing SKBR3 cells than WT Sindbis LV alone, while nonspecific gene transfer was reduced by ∼22-fold (∼13% of HER2^+^ cells versus ∼0.6% of HER2^−^ cells). These results underscore the enhanced selectivity and potent gene transfer seen using mSindbis LV redirected with bsIgG_1_^E2xHER2^.

**FIG 4:**
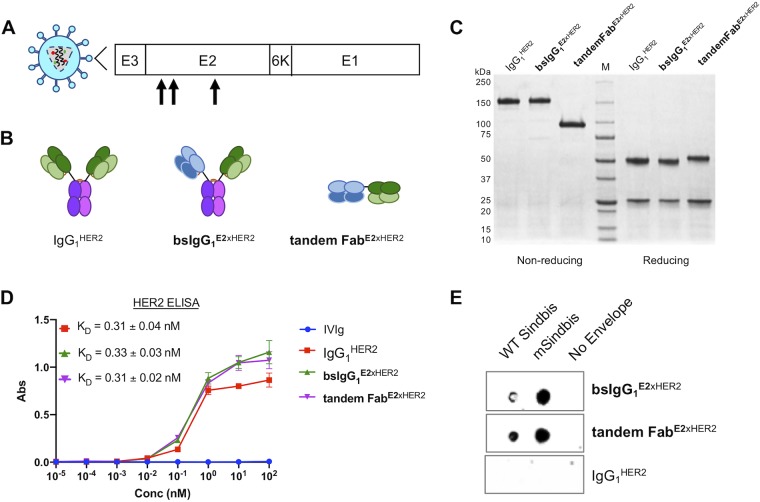
Characterization of bispecific tandem Fab. (A) Schematic representation of Sindbis glycoprotein domains E1 and E2. Mutated Sindbis envelope glycoprotein (mSindbis) contains mutations in the E2 domain (indicated by arrows) that ablate native receptor binding. E1 domain forms a heterodimer with E2, and E3 is a signal sequence peptide for E2 protein. (B) Schematic of control and bispecific Ab illustrating size and key design features between bsIgG_1_ and tandem Fab. (C) Nonreducing (left) and reducing (right) protein gels showing Coomassie blue staining of control and bispecific Ab. (D) Binding affinity of control and bispecific Ab to HER2-Fc chimera analyzed by ELISA. (E) Selective binding of bispecific Ab (bsIgG_1_ and tandem Fab) to Sindbis pseudotyped lentiviruses and absence of binding to negative control (no-envelope lentivirus) as visualized by dot blotting.

For *in vivo* applications, FcRn recycling and nonspecific uptake by Fc receptors on immune cells present a challenge with respect to the *in vivo* efficiency of targeted viral vectors via systemic administration. We thus evaluated an Fc-free tandem Fab that similarly binds Sindbis E2 and HER2 (Fig. 4A and B). The tandem Fab exhibited the expected molecular sizes as visualized on nonreduced and reduced protein gels (Fig. 4C). Using HER2-specific ELISAs, we found that tandem Fab^E2xHER2^ and bsIgG_1_^E2xHER2^ possessed binding affinities to HER2 comparable to those seen with the monoclonal IgG_1_^HER2^ control (Fig. 4D). We also verified via dot blotting the selective binding of tandem Fab to WT Sindbis and mSindbis-pseudotyped LV, and not to envelope-null LV (i.e., negative control). The negative antibody control, IgG_1_^HER2^, did not bind to WT Sindbis, mSindbis, or nonenveloped LV (Fig. 4E).

**FIG 5:**
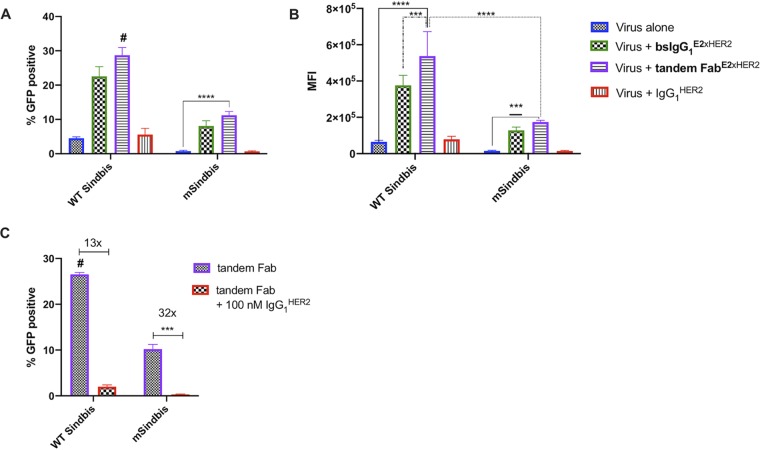
Comparable transduction efficiencies of targeted viruses coated with bsIgG_1_^E2xHER2^ and tandem Fab^E2xHER2^ in target HER2^+^ cells. Viral infectivity was measured by flow cytometry as (A) percentage of GFP-positive cells and (B) mean fluorescence intensity (MFI). (C) Targeted lentiviral infectivity is dependent upon HER2 specificity of bispecific antibody. Excess trastuzumab (IgG_1_^HER2^) substantially reduced viral infectivity of both targeted lentiviruses. All data represent results of *n* = 2 independent experiments (MOI = 3, [bsIgG_1_^E2xHER2^] = 1 nM, [tandem Fab^E2xHER2^] = 5 nM, and [IgG_1_^HER2^] = 1vnM) and were analyzed using two-way ANOVA with a *post hoc* Tukey’s test (#, *P* < 0.0001 [versus all conditions]; ****, *P* < 0.0001; ***, 0.0002 < *P* < 0.001; ***, *P* = 0.0003). For statistical analysis of group comparisons across all treatment groups, see Table S7 (A) and Table S8 (B and C).

Next, we compared the transduction efficiency of targeted LV with bsIgG_1_^E2xHER2^ to that of tandem Fab^E2xHER2^ using flow cytometry. As expected, bsIgG_1_^E2xHER2^ transduced ∼5-fold more SKBR3 cells than WT Sindbis LV and ∼10-fold more than mSindbis LV (Fig. 5A and B). Tandem Fab^E2xHER2^ also enhanced the transduction efficiency of WT Sindbis and mSindbis by ∼6-fold and ∼14-fold, respectively (Fig. 5A and B). At the tested bsAb concentrations, there was no statistical difference in transduction efficiency when LV was mixed with bsIgG_1_^E2xHER2^ or tandem Fab^E2xHER2^. BsIgG_1_^E2xHER2^ and tandem Fab^E2xHER2^ redirection was highly specific to HER2, as incubation with excess IgG_1_^HER2^ control efficiently blocked transduction, reducing the percentage of GFP^+^ cells (Fig. 5C). Overall, the transduction effectiveness facilitated by tandem Fab was similar to that seen with bsIgG_1_.

10.1128/mBio.02990-19.8 TABLE S7 Statistical comparison analysis results for Fig. 5A. Group comparisons were analyzed using two-way ANOVA and *post hoc* Tukey’s test. Download Table S7, DOCX file, 0.01 MB. Copyright © 2020 Parker et al. 2020 Parker et al. This is an open-access article distributed under the terms of the Creative Commons Attribution 4.0 International license.

10.1128/mBio.02990-19.9 TABLE S8 Statistical comparison analysis results for Fig. 5B and C. Group comparisons were analyzed using two-way ANOVA and *post hoc* Tukey’s test. Download Table S8, DOCX file, 0.01 MB. Copyright © 2020 Parker et al. 2020 Parker et al. This is an open-access article distributed under the terms of the Creative Commons Attribution 4.0 International license.

## DISCUSSION

Efficient and highly specific gene delivery *in vivo* continues to be a major challenge in human gene therapy. Despite their exceptional transduction potencies and potential for long-term transgene expression, the use of LV vectors *in vivo* remains very limited due to their poor cell and tissue specificity. WT Sindbis virus binds to the high-affinity laminin receptors (23) and heparin sulfate molecules (24) that are broadly distributed throughout the body, therefore making it a poor choice for targeted gene delivery *in vivo* when administered systemically. This broad native tropism of LV vectors has likely been responsible for the restriction of their use to *ex vivo* transduction, selection, and reinfusion into patients. Here, we showed that we can substantially improve the specificity of LV vectors by combining bsAbs that bind cell-specific receptors with abrogation of the native tropism of the LV vectors, culminating in a viral vector system that is 48 times more selective for HER2^+^ cells than for HER2^−^ cells.

The envelope of alphavirus Sindbis contains two integral membrane glycoproteins, E1 and E2. The E1 domain mediates endolysosomal membrane fusion and escape at low pH, whereas the E2 domain is responsible for binding to host cell receptor. Because receptor binding (attachment) and fusion (endosomal escape) are independent functions for Sindbis virus, it is possible to attenuate cell binding without hindering fusion. We found that bsAbs against Sindbis Env E2 domain were much more effective at transducing target cells than bsAbs that bind the Sindbis Env E1 domain. Since E1-binding bsAb was able to bind WT Sindbis and mSindbis LV, we hypothesize that bsIgG_1_^E1xHER2^ failed to mediate efficient transduction, possibly due to interference with the efficiencies of E1-mediated membrane fusion.

Multiple strategies have been implemented to confer new targeting specificities of mutated Sindbis pseudotyped lentiviruses using adaptors and antibodies (13, 17, 18, 20, 21, 25–27). As described previously, Morizono incorporated the ZZ domain of protein A into the Sindbis Env protein, thus enabling construction of a highly modular LV platform that can confer cell specificity simply by mixing with different monoclonal antibodies (MAbs) (13, 20, 25). This was demonstrated by the ∼15-fold selectivity for CD4^+^ cells over CD4^−^ cells resulting from simply mixing their viruses with anti-CD4 MAb (20). Despite the ease of its modular nature, the protein A-functionalized Sindbis system is vulnerable to competition from circulating serum immunoglobulins. Given the very high systemic IgG concentrations, circulating immunoglobulins may displace the targeting IgG_1_ from the protein A-functionalized Sindbis system, likely limiting its use for *in vivo* gene delivery (26). In contrast, our highly selective, targeted LV system (∼50-fold selectivity for HER2^+^ cells over HER2^−^ cells) relies on the use of bsAbs that bind to the E2 domain of Sindbis envelope; it is less vulnerable to competition from circulating serum immunoglobulins, supporting its use for direct *in vivo* delivery.

Chen and colleagues developed a redirection system based on covalent coupling of Sindbis pseudotyped lentiviruses and cell-specific adaptors that relied on the high-affinity, covalent pairing of bacterial proteins SpyTag and SpyCatcher. Use of their platform significantly reduced nonspecific cell binding and resulted in ∼7-fold selectivity for target cells over off-target cells (21). However, bacterium-based binding pairs such as SpyTag and SpyCatcher are likely immunogenic when introduced to the systemic circulation, which might induce an immune response leading to premature clearance of the vectors from the circulation and ultimately to lower transduction efficiency. The same group more recently pursued a similar approach by exploiting a disulfide bond-forming protein-peptide pair (PDZ1) and its pentapeptide ligand (TEFCA) to engineer a covalent pairing between PSZI-Sindbis pseudotyped lentivirus and cell-specific adaptor TEFCA. This stable targeting system exhibited ∼10-fold selectivity for target cells over off-target cells. A particular advantage of such covalent coupling is the ability to maintain transduction efficiency in the presence of human serum (21, 27).

Our bsAb-mSindbis LV system appeared to provide (∼50-fold) greater targeting selectivity than prior work that incorporated cell-specific targeting of modified LV. This greater specificity may be attributable to the specific Fab selected in our study possessing greater affinity to target receptors, as well as to reduced nonspecific binding due to the highly inert nature of Fabs. Another potential advantage of the use of human/humanized bsAb is a reduced likelihood of immune response and immunogenicity compared to the use of other protein/peptide cross-linkers. Unfortunately, a drawback to our current LV-bsAb system was the reduction in transduction efficiency as the serum content was increased beyond the 15% baseline (unpublished results). We anticipate that this shortcoming will be resolved by engineering bsAb with high affinity for the viral envelope using directed evolution strategies, such as phage display technology, leading to greater stability and resistance to serum sensitivity.

Adeno-associated virus (AAV) vectors remains the current gold standard in gene delivery, particularly in human patients, due to factors such as their simple binding and entry mechanisms, medium- to long-term gene expression depending on transduced cell type, and little risk of insertional mutagenesis. Not surprisingly, some investigators have exploited similar redirection strategies for AAV (28, 29). For instance, Gigout et al. incorporated additional amino acid changes into the capsid to reduce the native tropism of redirected AAV2 and observed that redirected AAV2 showed improved transduction efficiency with very low background infectivity (28). Similarly to our work here, Bartlett et al. used a bsAb to redirect WT AAV to target cells and reported a 70-fold improvement in gene transduction of target cells versus the use of WT AAV alone (29). However, the redirected bsAb-AAV system transduced off-target cells to levels comparable to those seen with target cells, underscoring a lack of targeting specificity that makes the system ill-suited to targeted gene delivery *in vivo*.

Despite its broad adoption in the gene therapy field, the use of AAV faces many challenges, including relatively modest transduction efficiency, lack of stable transgene integration, natural tropism to liver for off-target effects, and the need to use a very high MOI to achieve necessary transduction. These challenges are further exacerbated by the vector’s high immunogenicity as well as preexisting immunity, including preexisting neutralizing antibodies that limit the efficacy of initial and/or repeated treatment. In contrast, the general population has lower levels of preexisting Ab against LV (1). An added benefit of our approach is the use of human-based Fabs to redirect mSindbis LV to target cells; the “shield” of human Fab molecules around the mSindbis LV would likely block any preexisting and/or induced Ab from binding the LV, thereby preventing premature clearance from the circulation. Coupling those factors with the highly efficient transduction seen even at low MOI ratios and the potential for long-term transgene expression, we continue to believe LV-based gene vectors to represent an important alternative to AAV, depending on the specific *in vivo* gene therapy application.

In summary, a key challenge in gene therapy continues to be the lack of availability of a potent and yet highly selective gene vector system for select transduction of only targeted cells and tissues. By exploiting the specificity and ease of bsAb production with a mutated Sindbis pseudotyped LV vector that abrogates its native tropism, we have developed a system that is both efficient and specific. We showed that coupling bsAb with reduction of native tropism synergistically enhanced the selectivity of our targeted system. Our findings support bsAb-mSindbis LV vectors as a promising platform to enhance gene delivery to target cells/tissues *in vivo.*

## MATERIALS AND METHODS

### Cell lines.

293T cells were cultured in Dulbecco’s modified Eagle’s medium (DMEM) containing 10% fetal bovine serum (FBS). Human SKBR3 cells were purchased from the University of North Carolina at Chapel Hill (UNC-CH) Tissue Culture Facility, and A2780 cells were provided by Michael Jay (UNC-CH). SKBR3 cells were cultured in McCoy’s medium containing 15% FBS, and A2780 cells were cultured in RPMI 1640 containing 10% FBS and 1% l-glutamine. For coculture studies, both SKBR3 and A2780 cells were cultured in McCoy’s medium with 15% FBS. All cells were maintained at 37°C and 5% CO_2_.

### Preparation and characterization of fluorescent Sindbis pseudotyped lentivirus.

WT Sindbis and mSindbis pseudotyped lentiviruses (LV) were internally labeled with a GFP reporter gene. Particles were prepared by transfecting 293T cells with packaging plasmids pMDLg/pRRE and pRSV-Rev, transfer plasmid enhanced GFP (eGFP), and WT Sindbis or mSindbis envelope plasmid at a 1:1:1:1 ratio in serum-free media. The cell supernatant was collected 48 h later, and lentiviruses were purified from cell supernatant by ultracentrifugation using 25% (wt/vol) sucrose–HEPES-NaCl buffer. Lentiviruses were resuspended in 10% sucrose–HEPES-NaCl buffer, divided into aliquots, and stored at −80°C. Virus titer was quantified by the use of a quantitative PCR (qPCR)-based lentivirus titration kit according to the protocol of the manufacturer (Applied Biological Materials, Inc., Richmond, BC, Canada). Packaging plasmids pMDLg/pRRE (Addgene plasmid no. 12251) and pRSV-Rev (Addgene plasmid no. 12253) were provided by Didier Trono (30).

### Bispecific antibody construction and characterization.

Sequences for chimeric anti-Sindbis E1 or E2 and anti-HER2 antibodies (Ab) were generated by combining the V_H_/V_L_ regions of commercially available humanized anti-HER2 (trastuzumab) (22) and murine anti-Sindbis with the C_H_1/C_L_ and Fc regions of human IgG_1_ Ab. Mouse anti-Sindbis E1 and E2 V_H_/V_L_ sequences were provided by Diane Griffin (Johns Hopkins University; unpublished results). To generate bispecific IgG antibodies (bsIgG_1_) that recognized both Sindbis E1 or E2 and anti-HER2, separate orthogonal mutation sets were incorporated into anti-HER2 and anti-Sindbis Fab domains (31). Orthogonal mutation sets provided high-fidelity pairing of heavy and light chains; this technology was licensed through a partnership between Dualogics and UNC-CH. These mutations were also incorporated into the chimeric monoclonal antibody, IgG_1_^HER2^.

Plasmids encoding chimeric heavy and light chains were cotransfected into Expi293F cells (Thermo Fisher Scientific, Grand Island, NY) and grown for 72 h. IgG_1_^HER2^, bsIgG_1_^E2xHER2^, and bsIgG_1_^E1xHER2^ were purified from expression supernatant using protein A agarose (Thermo Fisher Scientific). BsIgG_1_ antibodies were separated via size exclusion chromatography (ENrich SEC 650 10 × 300 column; Bio-Rad Laboratories, Inc., Hercules, CA). The tandem Fab was designed to include a polyhistidine tag on its C terminus and was purified from expression supernatant using Ni-NTA agarose (Qiagen Inc, Germantown, MD). All purified antibodies were concentrated (Amicon Ultra; molecular weight cutoff [MWCO], 10 K) and subjected to buffer exchange into phosphate-buffered saline (PBS), concentrations were determined using *A*_280_ determinations (NanoDrop One/One), and the results were assessed for size and purity by sodium dodecyl sulfate-polyacrylamide gel electrophoresis (SDS-PAGE).

### Antibody binding affinity characterization.

HER2-specific ELISAs were performed to confirm binding of purified antibodies to HER2 as well as to compare dissociation constants of bispecific antibodies relative to the parental monoclonal control, IgG_1_^HER2^. Briefly, recombinant human ErbB2/HER2 Fc chimera protein (R&D Systems; catalog no. 1129-ER, Minneapolis, MN) was coated onto high-binding half-area 96-well Costar plates (Corning) at 1 μg/ml in bicarbonate buffer overnight at 4°C. After blocking of plates with 5% nonfat milk–PBS–0.05% Tween (PBST), purified antibody samples were diluted in 1% nonfat milk–PBST at various concentrations and incubated for 1 h, followed by washes with PBST. Bound antibodies were detected using goat anti-human kappa light-chain horseradish peroxidase (HRP) (Sigma-Aldrich; catalog no. A7164) (1:10,000 dilution) for 1 h followed by 1-step Ultra TMB (Thermo Fisher Scientific). After the HRP reaction was stopped with 2 N sulfuric acid, the absorbance at 450 nm and 570 nm was measured using a SpectraMax M2 plate reader (Molecular Devices).

To evaluate Sindbis specific binding, whole lentivirus (WT Sindbis, mSindbis, and no-envelope control) was blotted onto a nitrocellulose membrane. Bound bispecific antibodies were detected using goat anti-human kappa light-chain HRP (Sigma-Aldrich; catalog no. A7164) (1:10,000 dilution), followed by chemiluminescent detection using ECL reagents (Bio-Rad Laboratories, Inc.).

### Viral infectivity assay.

SKBR3 (HER2^+^) and A2780 (HER2^−^) cells were seeded at 3 × 10^4^ cells per well in a 96-well tissue culture-treated plate. Sindbis pseudotyped lentiviruses (multiplicity of infection [MOI] = 3) were premixed with antibodies at 1 nM concentration for 1 h at room temperature and were then incubated with cells at 37°C in 5% CO_2_. At 24 h later, the transduction mixture was removed from the cells and the cells were washed three times with PBS. Cells were allowed to grow for 72 h in fresh cell culture media at 37°C in 5% CO_2_. Cells were washed, and the percentage of transduced cells (GFP^+^) in each well was quantified using an iQue Screener Plus flow cytometer (Intellicyt, Albuquerque, NM). Additionally, to confirm that the viral infectivity was dependent upon the HER2 specificity of the bsAb, the viral infectivity assay was repeated with increasing concentrations of bsIgG_1_^E2xHER2^ in the presence and absence of excess IgG_1_^HER2^ (100 nM).

To test the selectivity of targeted viral systems for HER2^+^ cells, we established a coculture model of SKBR3 and A2780 cells that were maintained in McCoy’s 5A medium supplemented with 15% FBS. Cells in the coculture were infected with nontargeted or redirected LV vectors as described above. At 72 h postinfection, treated cells were washed and labeled with IgG_1_^HER2^ followed by goat anti-human IgG Alexa Fluor 594 (Thermo Fisher Scientific) to generate two key cell populations: cells doubly positive for GFP and HER2 expression and cells doubly negative for GFP and HER2 expression. The percentages of GFP^+^ cells among all HER2^+^ cells and of GFP^+^ cells among all HER2^−^ cells in each well were quantified using an iQue Screener PLUS flow cytometer. Data were analyzed using ForeCyt software and BD FACSDiva software.

### Statistical analysis.

All data are presented as means ± standard deviations (SD). All graphs and statistical tests were performed using GraphPad Prism 7 software. Group comparisons were analyzed using two-way analysis of variance (ANOVA) and a *post hoc* Tukey’s test. A *P* value of <0.05 was considered to indicate statistical significance.

10.1128/mBio.02990-19.10 TABLE S9 Statistical comparison analysis results for Fig. S1. Group comparisons were analyzed using two-way ANOVA and *post hoc* Tukey’s test. Download Table S9, DOCX file, 0.01 MB. Copyright © 2020 Parker et al. 2020 Parker et al. This is an open-access article distributed under the terms of the Creative Commons Attribution 4.0 International license.
