# Supplementary material for: Efficient and Highly Specific Gene Transfer Using Mutated Lentiviral Vectors Redirected with Bispecific Antibodies
Source: mBio. 2020 Jan 21;11(1):e02990-19. doi: 10.1128/mBio.02990-19 (PMC6989108; doi:10.1128/mBio.02990-19)
Supplement: TABLE S2 [file mBio.02990-19-st002.docx]

**Table S2**

| **Treatment Comparisons** | **Adjusted P Value** | **Summary** |
| --- | --- | --- |
| WT Sindbis:Virus alone vs. WT Sindbis:Virus + bsIgG_1_^E2xHER2^ | <0.0001 | **** |
| WT Sindbis:Virus alone vs. WT Sindbis:Virus + bsIgG_1_^E1xHER2^ | 0.9961 | ns |
| WT Sindbis:Virus alone vs. WT Sindbis:Virus + αHER2 IgG_1_ | >0.9999 | ns |
| WT Sindbis:Virus alone vs. mSindbis:Virus alone | 0.1156 | ns |
| WT Sindbis:Virus alone vs. mSindbis:Virus + bsIgG_1_^E2xHER2^ | <0.0001 | **** |
| WT Sindbis:Virus alone vs. mSindbis:Virus + bsIgG_1_^E1xHER2^ | 0.0618 | ns |
| WT Sindbis:Virus alone vs. mSindbis:Virus + αHER2 IgG_1_ | 0.0754 | ns |
| WT Sindbis:Virus + bsIgG_1_^E2xHER2^ vs. WT Sindbis:Virus + bsIgG_1_^E1xHER2^ | <0.0001 | **** |
| WT Sindbis:Virus + bsIgG_1_^E2xHER2^ vs. WT Sindbis:Virus + αHER2 IgG_1_ | <0.0001 | **** |
| WT Sindbis:Virus + bsIgG_1_^E2xHER2^ vs. mSindbis:Virus alone | <0.0001 | **** |
| WT Sindbis:Virus + bsIgG_1_^E2xHER2^ vs. mSindbis:Virus + bsIgG_1_^E2xHER2^ | <0.0001 | **** |
| WT Sindbis:Virus + bsIgG_1_^E2xHER2^ vs. mSindbis:Virus + bsIgG_1_^E1xHER2^ | <0.0001 | **** |
| WT Sindbis:Virus + bsIgG_1_^E2xHER2^ vs. mSindbis:Virus + αHER2 IgG_1_ | <0.0001 | **** |
| WT Sindbis:Virus + bsIgG_1_^E1xHER2^ vs. WT Sindbis:Virus + αHER2 IgG_1_ | 0.9979 | ns |
| WT Sindbis:Virus + bsIgG_1_^E1xHER2^ vs. mSindbis:Virus alone | 0.4133 | ns |
| WT Sindbis:Virus + bsIgG_1_^E1xHER2^ vs. mSindbis:Virus + bsIgG_1_^E2xHER2^ | <0.0001 | **** |
| WT Sindbis:Virus + bsIgG_1_^E1xHER2^ vs. mSindbis:Virus + bsIgG_1_^E1xHER2^ | 0.2759 | ns |
| WT Sindbis:Virus + bsIgG_1_^E1xHER2^ vs. mSindbis:Virus + αHER2 IgG_1_ | 0.3083 | ns |
| WT Sindbis:Virus + αHER2 IgG_1_ vs. mSindbis:Virus alone | 0.1353 | ns |
| WT Sindbis:Virus + αHER2 IgG_1_ vs. mSindbis:Virus + bsIgG_1_^E2xHER2^ | <0.0001 | **** |
| WT Sindbis:Virus + αHER2 IgG_1_ vs. mSindbis:Virus + bsIgG_1_^E1xHER2^ | 0.0741 | ns |
| WT Sindbis:Virus + αHER2 IgG_1_ vs. mSindbis:Virus + αHER2 IgG_1_ | 0.0894 | ns |
| mSindbis:Virus alone vs. mSindbis:Virus + bsIgG_1_^E2xHER2^ | <0.0001 | **** |
| mSindbis:Virus alone vs. mSindbis:Virus + bsIgG_1_^E1xHER2^ | >0.9999 | ns |
| mSindbis:Virus alone vs. mSindbis:Virus + αHER2 IgG_1_ | >0.9999 | ns |
| mSindbis:Virus + bsIgG_1_^E2xHER2^ vs. mSindbis:Virus + bsIgG_1_^E1xHER2^ | <0.0001 | **** |
| mSindbis:Virus + bsIgG_1_^E2xHER2^ vs. mSindbis:Virus + αHER2 IgG_1_ | <0.0001 | **** |
| mSindbis:Virus + bsIgG_1_^E1xHER2^ vs. mSindbis:Virus + αHER2 IgG_1_ | >0.9999 | ns |
