# Supplementary material for: Efficient and Highly Specific Gene Transfer Using Mutated Lentiviral Vectors Redirected with Bispecific Antibodies
Source: mBio. 2020 Jan 21;11(1):e02990-19. doi: 10.1128/mBio.02990-19 (PMC6989108; doi:10.1128/mBio.02990-19)
Supplement: TABLE S3 [file mBio.02990-19-st003.docx]

**Table S3**

| **WT Sindbis Treatment Comparisons** | **Adjusted P Value** | **Summary** |
| --- | --- | --- |
| HER2^-^ cells only:Virus alone vs. HER2^-^ cells only:Virus + αE2 x αHER2 bsIgG_1_ | 0.9816 | ns |
| HER2^-^ cells only:Virus alone vs. HER2^-^ cells only:Virus + αE1 x αHER2 bsIgG_1_ | >0.9999 | ns |
| HER2^-^ cells only:Virus alone vs. HER2^-^ cells only:Virus + αHER2 IgG_1_ | >0.9999 | ns |
| HER2^-^ cells only:Virus alone vs. HER2^+^ cells only:Virus alone | 0.7636 | ns |
| HER2^-^ cells only:Virus alone vs. HER2^+^ cells only:Virus + αE2 x αHER2 bsIgG_1_ | <0.0001 | **** |
| HER2^-^ cells only:Virus alone vs. HER2^+^ cells only:Virus + αE1 x αHER2 bsIgG_1_ | 0.9857 | ns |
| HER2^-^ cells only:Virus alone vs. HER2^+^ cells only:Virus + αHER2 IgG_1_ | 0.5437 | ns |
| HER2^-^ cells only:Virus + αE2 x αHER2 bsIgG_1_ vs. HER2^-^ cells only:Virus + αE1 x αHER2 bsIgG_1_ | 0.9934 | ns |
| HER2^-^ cells only:Virus + αE2 x αHER2 bsIgG_1_ vs. HER2^-^ cells only:Virus + αHER2 IgG_1_ | 0.9855 | ns |
| HER2^-^ cells only:Virus + αE2 x αHER2 bsIgG_1_ vs. HER2^+^ cells only:Virus alone | 0.9975 | ns |
| HER2^-^ cells only:Virus + αE2 x αHER2 bsIgG_1_ vs. HER2^+^ cells only:Virus + αE2 x αHER2 bsIgG_1_ | <0.0001 | **** |
| HER2^-^ cells only:Virus + αE2 x αHER2 bsIgG_1_ vs. HER2^+^ cells only:Virus + αE1 x αHER2 bsIgG_1_ | >0.9999 | ns |
| HER2^-^ cells only:Virus + αE2 x αHER2 bsIgG_1_ vs. HER2^+^ cells only:Virus + αHER2 IgG_1_ | 0.967 | ns |
| HER2^-^ cells only:Virus + αE1 x αHER2 bsIgG_1_ vs. HER2^-^ cells only:Virus + αHER2 IgG_1_ | >0.9999 | ns |
| HER2^-^ cells only:Virus + αE1 x αHER2 bsIgG_1_ vs. HER2^+^ cells only:Virus alone | 0.8394 | ns |
| HER2^-^ cells only:Virus + αE1 x αHER2 bsIgG_1_ vs. HER2^+^ cells only:Virus + αE2 x αHER2 bsIgG_1_ | <0.0001 | **** |
| HER2^-^ cells only:Virus + αE1 x αHER2 bsIgG_1_ vs. HER2^+^ cells only:Virus + αE1 x αHER2 bsIgG_1_ | 0.9952 | ns |
| HER2^-^ cells only:Virus + αE1 x αHER2 bsIgG_1_ vs. HER2^+^ cells only:Virus + αHER2 IgG_1_ | 0.6359 | ns |
| HER2^-^ cells only:Virus + αHER2 IgG_1_ vs. HER2^+^ cells only:Virus alone | 0.7838 | ns |
| HER2^-^ cells only:Virus + αHER2 IgG_1_ vs. HER2^+^ cells only:Virus + αE2 x αHER2 bsIgG_1_ | <0.0001 | **** |
| HER2^-^ cells only:Virus + αHER2 IgG_1_ vs. HER2^+^ cells only:Virus + αE1 x αHER2 bsIgG_1_ | 0.9889 | ns |
| HER2^-^ cells only:Virus + αHER2 IgG_1_ vs. HER2^+^ cells only:Virus + αHER2 IgG_1_ | 0.5668 | ns |
| HER2^+^ cells only:Virus alone vs. HER2^+^ cells only:Virus + αE2 x αHER2 bsIgG_1_ | <0.0001 | **** |
| HER2^+^ cells only:Virus alone vs. HER2^+^ cells only:Virus + αE1 x αHER2 bsIgG_1_ | 0.9964 | ns |
| HER2^+^ cells only:Virus alone vs. HER2^+^ cells only:Virus + αHER2 IgG_1_ | >0.9999 | ns |
| HER2^+^ cells only:Virus + αE2 x αHER2 bsIgG_1_ vs. HER2^+^ cells only:Virus + αE1 x αHER2 bsIgG_1_ | <0.0001 | **** |
| HER2^+^ cells only:Virus + αE2 x αHER2 bsIgG_1_ vs. HER2^+^ cells only:Virus + αHER2 IgG_1_ | <0.0001 | **** |
| HER2^+^ cells only:Virus + αE1 x αHER2 bsIgG_1_ vs. HER2^+^ cells only:Virus + αHER2 IgG_1_ | 0.9597 | ns |
| **mSindbis Treatment Comparisons** | **Adjusted P Value** | **Summary** |
| HER2^-^ cells only:Virus alone vs. HER2^-^ cells only:Virus + αE2 x αHER2 bsIgG_1_ | >0.9999 | ns |
| HER2^-^ cells only:Virus alone vs. HER2^-^ cells only:Virus + αE1 x αHER2 bsIgG_1_ | >0.9999 | ns |
| HER2^-^ cells only:Virus alone vs. HER2^-^ cells only:Virus + αHER2 IgG_1_ | >0.9999 | ns |
| HER2^-^ cells only:Virus alone vs. HER2^+^ cells only:Virus alone | 0.4556 | ns |
| HER2^-^ cells only:Virus alone vs. HER2^+^ cells only:Virus + αE2 x αHER2 bsIgG_1_ | <0.0001 | **** |
| HER2^-^ cells only:Virus alone vs. HER2^+^ cells only:Virus + αE1 x αHER2 bsIgG_1_ | 0.4055 | ns |
| HER2^-^ cells only:Virus alone vs. HER2^+^ cells only:Virus + αHER2 IgG_1_ | 0.6115 | ns |
| HER2^-^ cells only:Virus + αE2 x αHER2 bsIgG_1_ vs. HER2^-^ cells only:Virus + αE1 x αHER2 bsIgG_1_ | >0.9999 | ns |
| HER2^-^ cells only:Virus + αE2 x αHER2 bsIgG_1_ vs. HER2^-^ cells only:Virus + αHER2 IgG_1_ | >0.9999 | ns |
| HER2^-^ cells only:Virus + αE2 x αHER2 bsIgG_1_ vs. HER2^+^ cells only:Virus alone | 0.5298 | ns |
| HER2^-^ cells only:Virus + αE2 x αHER2 bsIgG_1_ vs. HER2^+^ cells only:Virus + αE2 x αHER2 bsIgG_1_ | <0.0001 | **** |
| HER2^-^ cells only:Virus + αE2 x αHER2 bsIgG_1_ vs. HER2^+^ cells only:Virus + αE1 x αHER2 bsIgG_1_ | 0.4768 | ns |
| HER2^-^ cells only:Virus + αE2 x αHER2 bsIgG_1_ vs. HER2^+^ cells only:Virus + αHER2 IgG_1_ | 0.687 | ns |
| HER2^-^ cells only:Virus + αE1 x αHER2 bsIgG_1_ vs. HER2^-^ cells only:Virus + αHER2 IgG_1_ | >0.9999 | ns |
| HER2^-^ cells only:Virus + αE1 x αHER2 bsIgG_1_ vs. HER2^+^ cells only:Virus alone | 0.4106 | ns |
| HER2^-^ cells only:Virus + αE1 x αHER2 bsIgG_1_ vs. HER2^+^ cells only:Virus + αE2 x αHER2 bsIgG_1_ | <0.0001 | **** |
| HER2^-^ cells only:Virus + αE1 x αHER2 bsIgG_1_ vs. HER2^+^ cells only:Virus + αE1 x αHER2 bsIgG_1_ | 0.363 | ns |
| HER2^-^ cells only:Virus + αE1 x αHER2 bsIgG_1_ vs. HER2^+^ cells only:Virus + αHER2 IgG_1_ | 0.5629 | ns |
| HER2^-^ cells only:Virus + αHER2 IgG_1_ vs. HER2^+^ cells only:Virus alone | 0.4575 | ns |
| HER2^-^ cells only:Virus + αHER2 IgG_1_ vs. HER2^+^ cells only:Virus + αE2 x αHER2 bsIgG_1_ | <0.0001 | **** |
| HER2^-^ cells only:Virus + αHER2 IgG_1_ vs. HER2^+^ cells only:Virus + αE1 x αHER2 bsIgG_1_ | 0.4074 | ns |
| HER2^-^ cells only:Virus + αHER2 IgG_1_ vs. HER2^+^ cells only:Virus + αHER2 IgG_1_ | 0.6135 | ns |
| HER2^+^ cells only:Virus alone vs. HER2^+^ cells only:Virus + αE2 x αHER2 bsIgG_1_ | <0.0001 | **** |
| HER2^+^ cells only:Virus alone vs. HER2^+^ cells only:Virus + αE1 x αHER2 bsIgG_1_ | >0.9999 | ns |
| HER2^+^ cells only:Virus alone vs. HER2^+^ cells only:Virus + αHER2 IgG_1_ | >0.9999 | ns |
| HER2^+^ cells only:Virus + αE2 x αHER2 bsIgG_1_ vs. HER2^+^ cells only:Virus + αE1 x αHER2 bsIgG_1_ | <0.0001 | **** |
| HER2^+^ cells only:Virus + αE2 x αHER2 bsIgG_1_ vs. HER2^+^ cells only:Virus + αHER2 IgG_1_ | <0.0001 | **** |
| HER2^+^ cells only:Virus + αE1 x αHER2 bsIgG_1_ vs. HER2^+^ cells only:Virus + αHER2 IgG_1_ | >0.9999 | ns |
