# Supplementary material for: Efficient and Highly Specific Gene Transfer Using Mutated Lentiviral Vectors Redirected with Bispecific Antibodies
Source: mBio. 2020 Jan 21;11(1):e02990-19. doi: 10.1128/mBio.02990-19 (PMC6989108; doi:10.1128/mBio.02990-19)
Supplement: TABLE S4 [file mBio.02990-19-st004.docx]

**Table S4**

| **WT Sindbis Treatment Comparisons** | **Adjusted P Value** | **Summary** |
| --- | --- | --- |
| HER2^-^ cells only:Virus alone vs. HER2^-^ cells only:Virus + αE2 x αHER2 bsIgG_1_ | >0.9999 | ns |
| HER2^-^ cells only:Virus alone vs. HER2^-^ cells only:Virus + αE1 x αHER2 bsIgG_1_ | >0.9999 | ns |
| HER2^-^ cells only:Virus alone vs. HER2^-^ cells only:Virus + αHER2 IgG_1_ | >0.9999 | ns |
| HER2^-^ cells only:Virus alone vs. HER2^+^ cells only:Virus alone | 0.2259 | ns |
| HER2^-^ cells only:Virus alone vs. HER2^+^ cells only:Virus + αE2 x αHER2 bsIgG_1_ | <0.0001 | **** |
| HER2^-^ cells only:Virus alone vs. HER2^+^ cells only:Virus + αE1 x αHER2 bsIgG_1_ | 0.7961 | ns |
| HER2^-^ cells only:Virus alone vs. HER2^+^ cells only:Virus + αHER2 IgG_1_ | 0.0109 | * |
| HER2^-^ cells only:Virus + αE2 x αHER2 bsIgG_1_ vs. HER2^-^ cells only:Virus + αE1 x αHER2 bsIgG_1_ | >0.9999 | ns |
| HER2^-^ cells only:Virus + αE2 x αHER2 bsIgG_1_ vs. HER2^-^ cells only:Virus + αHER2 IgG_1_ | >0.9999 | ns |
| HER2^-^ cells only:Virus + αE2 x αHER2 bsIgG_1_ vs. HER2^+^ cells only:Virus alone | 0.2531 | ns |
| HER2^-^ cells only:Virus + αE2 x αHER2 bsIgG_1_ vs. HER2^+^ cells only:Virus + αE2 x αHER2 bsIgG_1_ | <0.0001 | **** |
| HER2^-^ cells only:Virus + αE2 x αHER2 bsIgG_1_ vs. HER2^+^ cells only:Virus + αE1 x αHER2 bsIgG_1_ | 0.8298 | ns |
| HER2^-^ cells only:Virus + αE2 x αHER2 bsIgG_1_ vs. HER2^+^ cells only:Virus + αHER2 IgG_1_ | 0.0128 | * |
| HER2^-^ cells only:Virus + αE1 x αHER2 bsIgG_1_ vs. HER2^-^ cells only:Virus + αHER2 IgG_1_ | >0.9999 | ns |
| HER2^-^ cells only:Virus + αE1 x αHER2 bsIgG_1_ vs. HER2^+^ cells only:Virus alone | 0.2351 | ns |
| HER2^-^ cells only:Virus + αE1 x αHER2 bsIgG_1_ vs. HER2^+^ cells only:Virus + αE2 x αHER2 bsIgG_1_ | <0.0001 | **** |
| HER2^-^ cells only:Virus + αE1 x αHER2 bsIgG_1_ vs. HER2^+^ cells only:Virus + αE1 x αHER2 bsIgG_1_ | 0.8081 | ns |
| HER2^-^ cells only:Virus + αE1 x αHER2 bsIgG_1_ vs. HER2^+^ cells only:Virus + αHER2 IgG_1_ | 0.0116 | * |
| HER2^-^ cells only:Virus + αHER2 IgG_1_ vs. HER2^+^ cells only:Virus alone | 0.2155 | ns |
| HER2^-^ cells only:Virus + αHER2 IgG_1_ vs. HER2^+^ cells only:Virus + αE2 x αHER2 bsIgG_1_ | <0.0001 | **** |
| HER2^-^ cells only:Virus + αHER2 IgG_1_ vs. HER2^+^ cells only:Virus + αE1 x αHER2 bsIgG_1_ | 0.7816 | ns |
| HER2^-^ cells only:Virus + αHER2 IgG_1_ vs. HER2^+^ cells only:Virus + αHER2 IgG_1_ | 0.0102 | * |
| HER2^+^ cells only:Virus alone vs. HER2^+^ cells only:Virus + αE2 x αHER2 bsIgG_1_ | <0.0001 | **** |
| HER2^+^ cells only:Virus alone vs. HER2^+^ cells only:Virus + αE1 x αHER2 bsIgG_1_ | 0.9641 | ns |
| HER2^+^ cells only:Virus alone vs. HER2^+^ cells only:Virus + αHER2 IgG_1_ | 0.8296 | ns |
| HER2^+^ cells only:Virus + αE2 x αHER2 bsIgG_1_ vs. HER2^+^ cells only:Virus + αE1 x αHER2 bsIgG_1_ | <0.0001 | **** |
| HER2^+^ cells only:Virus + αE2 x αHER2 bsIgG_1_ vs. HER2^+^ cells only:Virus + αHER2 IgG_1_ | <0.0001 | **** |
| HER2^+^ cells only:Virus + αE1 x αHER2 bsIgG_1_ vs. HER2^+^ cells only:Virus + αHER2 IgG_1_ | 0.2529 | ns |
| **mSindbis Treatment Comparisons** | **Adjusted P Value** | **Summary** |
| HER2^-^ cells only:Virus alone vs. HER2^-^ cells only:Virus + αE2 x αHER2 bsIgG_1_ | >0.9999 | ns |
| HER2^-^ cells only:Virus alone vs. HER2^-^ cells only:Virus + αE1 x αHER2 bsIgG_1_ | >0.9999 | ns |
| HER2^-^ cells only:Virus alone vs. HER2^-^ cells only:Virus + αHER2 IgG_1_ | >0.9999 | ns |
| HER2^-^ cells only:Virus alone vs. HER2^+^ cells only:Virus alone | 0.9998 | ns |
| HER2^-^ cells only:Virus alone vs. HER2^+^ cells only:Virus + αE2 x αHER2 bsIgG_1_ | <0.0001 | **** |
| HER2^-^ cells only:Virus alone vs. HER2^+^ cells only:Virus + αE1 x αHER2 bsIgG_1_ | 0.9983 | ns |
| HER2^-^ cells only:Virus alone vs. HER2^+^ cells only:Virus + αHER2 IgG_1_ | 0.998 | ns |
| HER2^-^ cells only:Virus + αE2 x αHER2 bsIgG_1_ vs. HER2^-^ cells only:Virus + αE1 x αHER2 bsIgG_1_ | >0.9999 | ns |
| HER2^-^ cells only:Virus + αE2 x αHER2 bsIgG_1_ vs. HER2^-^ cells only:Virus + αHER2 IgG_1_ | >0.9999 | ns |
| HER2^-^ cells only:Virus + αE2 x αHER2 bsIgG_1_ vs. HER2^+^ cells only:Virus alone | 0.9999 | ns |
| HER2^-^ cells only:Virus + αE2 x αHER2 bsIgG_1_ vs. HER2^+^ cells only:Virus + αE2 x αHER2 bsIgG_1_ | <0.0001 | **** |
| HER2^-^ cells only:Virus + αE2 x αHER2 bsIgG_1_ vs. HER2^+^ cells only:Virus + αE1 x αHER2 bsIgG_1_ | 0.9986 | ns |
| HER2^-^ cells only:Virus + αE2 x αHER2 bsIgG_1_ vs. HER2^+^ cells only:Virus + αHER2 IgG_1_ | 0.9983 | ns |
| HER2^-^ cells only:Virus + αE1 x αHER2 bsIgG_1_ vs. HER2^-^ cells only:Virus + αHER2 IgG_1_ | >0.9999 | ns |
| HER2^-^ cells only:Virus + αE1 x αHER2 bsIgG_1_ vs. HER2^+^ cells only:Virus alone | 0.9998 | ns |
| HER2^-^ cells only:Virus + αE1 x αHER2 bsIgG_1_ vs. HER2^+^ cells only:Virus + αE2 x αHER2 bsIgG_1_ | <0.0001 | **** |
| HER2^-^ cells only:Virus + αE1 x αHER2 bsIgG_1_ vs. HER2^+^ cells only:Virus + αE1 x αHER2 bsIgG_1_ | 0.9986 | ns |
| HER2^-^ cells only:Virus + αE1 x αHER2 bsIgG_1_ vs. HER2^+^ cells only:Virus + αHER2 IgG_1_ | 0.9983 | ns |
| HER2^-^ cells only:Virus + αHER2 IgG_1_ vs. HER2^+^ cells only:Virus alone | 0.9998 | ns |
| HER2^-^ cells only:Virus + αHER2 IgG_1_ vs. HER2^+^ cells only:Virus + αE2 x αHER2 bsIgG_1_ | <0.0001 | **** |
| HER2^-^ cells only:Virus + αHER2 IgG_1_ vs. HER2^+^ cells only:Virus + αE1 x αHER2 bsIgG_1_ | 0.9984 | ns |
| HER2^-^ cells only:Virus + αHER2 IgG_1_ vs. HER2^+^ cells only:Virus + αHER2 IgG_1_ | 0.9981 | ns |
| HER2^+^ cells only:Virus alone vs. HER2^+^ cells only:Virus + αE2 x αHER2 bsIgG_1_ | <0.0001 | **** |
| HER2^+^ cells only:Virus alone vs. HER2^+^ cells only:Virus + αE1 x αHER2 bsIgG_1_ | >0.9999 | ns |
| HER2^+^ cells only:Virus alone vs. HER2^+^ cells only:Virus + αHER2 IgG_1_ | >0.9999 | ns |
| HER2^+^ cells only:Virus + αE2 x αHER2 bsIgG_1_ vs. HER2^+^ cells only:Virus + αE1 x αHER2 bsIgG_1_ | <0.0001 | **** |
| HER2^+^ cells only:Virus + αE2 x αHER2 bsIgG_1_ vs. HER2^+^ cells only:Virus + αHER2 IgG_1_ | <0.0001 | **** |
| HER2^+^ cells only:Virus + αE1 x αHER2 bsIgG_1_ vs. HER2^+^ cells only:Virus + αHER2 IgG_1_ | >0.9999 | ns |
