# Supplementary material for: Efficient and Highly Specific Gene Transfer Using Mutated Lentiviral Vectors Redirected with Bispecific Antibodies
Source: mBio. 2020 Jan 21;11(1):e02990-19. doi: 10.1128/mBio.02990-19 (PMC6989108; doi:10.1128/mBio.02990-19)
Supplement: TABLE S5 [file mBio.02990-19-st005.docx]

**Table S5**

| **Treatment Comparisons** | **Adjusted P Value** | **Summary** |
| --- | --- | --- |
| WT Sindbis:Virus alone vs. WT Sindbis:Virus + αE2 x αHER2 bsIgG_1_ | <0.0001 | **** |
| WT Sindbis:Virus alone vs. WT Sindbis:Virus + αE1 x αHER2 bsIgG_1_ | >0.9999 | ns |
| WT Sindbis:Virus alone vs. WT Sindbis:Virus + αHER2 IgG_1_ | >0.9999 | ns |
| WT Sindbis:Virus alone vs. mSindbis:Virus alone | 0.1602 | ns |
| WT Sindbis:Virus alone vs. mSindbis:Virus + αE2 x αHER2 bsIgG_1_ | 0.4125 | ns |
| WT Sindbis:Virus alone vs. mSindbis:Virus + αE1 x αHER2 bsIgG_1_ | 0.1524 | ns |
| WT Sindbis:Virus alone vs. mSindbis:Virus + αHER2 IgG_1_ | 0.1576 | ns |
| WT Sindbis:Virus + αE2 x αHER2 bsIgG_1_ vs. WT Sindbis:Virus + αE1 x αHER2 bsIgG_1_ | <0.0001 | **** |
| WT Sindbis:Virus + αE2 x αHER2 bsIgG_1_ vs. WT Sindbis:Virus + αHER2 IgG_1_ | <0.0001 | **** |
| WT Sindbis:Virus + αE2 x αHER2 bsIgG_1_ vs. mSindbis:Virus alone | <0.0001 | **** |
| WT Sindbis:Virus + αE2 x αHER2 bsIgG_1_ vs. mSindbis:Virus + αE2 x αHER2 bsIgG_1_ | <0.0001 | **** |
| WT Sindbis:Virus + αE2 x αHER2 bsIgG_1_ vs. mSindbis:Virus + αE1 x αHER2 bsIgG_1_ | <0.0001 | **** |
| WT Sindbis:Virus + αE2 x αHER2 bsIgG_1_ vs. mSindbis:Virus + αHER2 IgG_1_ | <0.0001 | **** |
| WT Sindbis:Virus + αE1 x αHER2 bsIgG_1_ vs. WT Sindbis:Virus + αHER2 IgG_1_ | 0.9979 | ns |
| WT Sindbis:Virus + αE1 x αHER2 bsIgG_1_ vs. mSindbis:Virus alone | 0.2509 | ns |
| WT Sindbis:Virus + αE1 x αHER2 bsIgG_1_ vs. mSindbis:Virus + αE2 x αHER2 bsIgG_1_ | 0.2822 | ns |
| WT Sindbis:Virus + αE1 x αHER2 bsIgG_1_ vs. mSindbis:Virus + αE1 x αHER2 bsIgG_1_ | 0.2398 | ns |
| WT Sindbis:Virus + αE1 x αHER2 bsIgG_1_ vs. mSindbis:Virus + αHER2 IgG_1_ | 0.2472 | ns |
| WT Sindbis:Virus + αHER2 IgG_1_ vs. mSindbis:Virus alone | 0.0755 | ns |
| WT Sindbis:Virus + αHER2 IgG_1_ vs. mSindbis:Virus + αE2 x αHER2 bsIgG_1_ | 0.6445 | ns |
| WT Sindbis:Virus + αHER2 IgG_1_ vs. mSindbis:Virus + αE1 x αHER2 bsIgG_1_ | 0.0714 | ns |
| WT Sindbis:Virus + αHER2 IgG_1_ vs. mSindbis:Virus + αHER2 IgG_1_ | 0.0742 | ns |
| mSindbis:Virus alone vs. mSindbis:Virus + αE2 x αHER2 bsIgG_1_ | 0.0012 | ** |
| mSindbis:Virus alone vs. mSindbis:Virus + αE1 x αHER2 bsIgG_1_ | >0.9999 | ns |
| mSindbis:Virus alone vs. mSindbis:Virus + αHER2 IgG_1_ | >0.9999 | ns |
| mSindbis:Virus + αE2 x αHER2 bsIgG_1_ vs. mSindbis:Virus + αE1 x αHER2 bsIgG_1_ | 0.0012 | ** |
| mSindbis:Virus + αE2 x αHER2 bsIgG_1_ vs. mSindbis:Virus + αHER2 IgG_1_ | 0.0012 | ** |
| mSindbis:Virus + αE1 x αHER2 bsIgG_1_ vs. mSindbis:Virus + αHER2 IgG_1_ | >0.9999 | ns |
