# Supplementary material for: Efficient and Highly Specific Gene Transfer Using Mutated Lentiviral Vectors Redirected with Bispecific Antibodies
Source: mBio. 2020 Jan 21;11(1):e02990-19. doi: 10.1128/mBio.02990-19 (PMC6989108; doi:10.1128/mBio.02990-19)
Supplement: TABLE S6 [file mBio.02990-19-st006.docx]

**Table S6**

| **Treatment Comparisons** | **Adjusted P Value** | **Summary** |
| --- | --- | --- |
| WT Sindbis:Virus alone vs. WT Sindbis:Virus + αE2 x αHER2 bsIgG_1_ | 0.025 | * |
| WT Sindbis:Virus alone vs. WT Sindbis:Virus + αE1 x αHER2 bsIgG_1_ | 0.8375 | ns |
| WT Sindbis:Virus alone vs. WT Sindbis:Virus + αHER2 IgG_1_ | 0.8734 | ns |
| WT Sindbis:Virus alone vs. mSindbis:Virus alone | <0.0001 | **** |
| WT Sindbis:Virus alone vs. mSindbis:Virus + αE2 x αHER2 bsIgG_1_ | <0.0001 | **** |
| WT Sindbis:Virus alone vs. mSindbis:Virus + αE1 x αHER2 bsIgG_1_ | <0.0001 | **** |
| WT Sindbis:Virus alone vs. mSindbis:Virus + αHER2 IgG_1_ | <0.0001 | **** |
| WT Sindbis:Virus + αE2 x αHER2 bsIgG_1_ vs. WT Sindbis:Virus + αE1 x αHER2 bsIgG_1_ | 0.3854 | ns |
| WT Sindbis:Virus + αE2 x αHER2 bsIgG_1_ vs. WT Sindbis:Virus + αHER2 IgG_1_ | 0.3421 | ns |
| WT Sindbis:Virus + αE2 x αHER2 bsIgG_1_ vs. mSindbis:Virus alone | <0.0001 | **** |
| WT Sindbis:Virus + αE2 x αHER2 bsIgG_1_ vs. mSindbis:Virus + αE2 x αHER2 bsIgG_1_ | <0.0001 | **** |
| WT Sindbis:Virus + αE2 x αHER2 bsIgG_1_ vs. mSindbis:Virus + αE1 x αHER2 bsIgG_1_ | <0.0001 | **** |
| WT Sindbis:Virus + αE2 x αHER2 bsIgG_1_ vs. mSindbis:Virus + αHER2 IgG_1_ | <0.0001 | **** |
| WT Sindbis:Virus + αE1 x αHER2 bsIgG_1_ vs. WT Sindbis:Virus + αHER2 IgG_1_ | >0.9999 | ns |
| WT Sindbis:Virus + αE1 x αHER2 bsIgG_1_ vs. mSindbis:Virus alone | <0.0001 | **** |
| WT Sindbis:Virus + αE1 x αHER2 bsIgG_1_ vs. mSindbis:Virus + αE2 x αHER2 bsIgG_1_ | <0.0001 | **** |
| WT Sindbis:Virus + αE1 x αHER2 bsIgG_1_ vs. mSindbis:Virus + αE1 x αHER2 bsIgG_1_ | <0.0001 | **** |
| WT Sindbis:Virus + αE1 x αHER2 bsIgG_1_ vs. mSindbis:Virus + αHER2 IgG_1_ | <0.0001 | **** |
| WT Sindbis:Virus + αHER2 IgG_1_ vs. mSindbis:Virus alone | <0.0001 | **** |
| WT Sindbis:Virus + αHER2 IgG_1_ vs. mSindbis:Virus + αE2 x αHER2 bsIgG_1_ | <0.0001 | **** |
| WT Sindbis:Virus + αHER2 IgG_1_ vs. mSindbis:Virus + αE1 x αHER2 bsIgG_1_ | <0.0001 | **** |
| WT Sindbis:Virus + αHER2 IgG_1_ vs. mSindbis:Virus + αHER2 IgG_1_ | <0.0001 | **** |
| mSindbis:Virus alone vs. mSindbis:Virus + αE2 x αHER2 bsIgG_1_ | 0.9999 | ns |
| mSindbis:Virus alone vs. mSindbis:Virus + αE1 x αHER2 bsIgG_1_ | >0.9999 | ns |
| mSindbis:Virus alone vs. mSindbis:Virus + αHER2 IgG_1_ | >0.9999 | ns |
| mSindbis:Virus + αE2 x αHER2 bsIgG_1_ vs. mSindbis:Virus + αE1 x αHER2 bsIgG_1_ | 0.9998 | ns |
| mSindbis:Virus + αE2 x αHER2 bsIgG_1_ vs. mSindbis:Virus + αHER2 IgG_1_ | >0.9999 | ns |
| mSindbis:Virus + αE1 x αHER2 bsIgG_1_ vs. mSindbis:Virus + αHER2 IgG_1_ | >0.9999 | ns |
