# Supplementary material for: Efficient and Highly Specific Gene Transfer Using Mutated Lentiviral Vectors Redirected with Bispecific Antibodies
Source: mBio. 2020 Jan 21;11(1):e02990-19. doi: 10.1128/mBio.02990-19 (PMC6989108; doi:10.1128/mBio.02990-19)
Supplement: TABLE S7 [file mBio.02990-19-st007.docx]

**Table S7**

| **Treatment Comparisons** | **Adjusted P Value** | **Summary** |
| --- | --- | --- |
| WT Sindbis:Virus alone vs. WT Sindbis:bsIgG_1_**^E2^**^xHER2^ | <0.0001 | **** |
| WT Sindbis:Virus alone vs. WT Sindbis:tandem Fab**^E2^**^xHER2^ | <0.0001 | **** |
| WT Sindbis:Virus alone vs. WT Sindbis:Virus + IgG_1_^HER2^ | 0.9404 | ns |
| WT Sindbis:Virus alone vs. mSindbis:Virus alone | 0.0031 | ** |
| WT Sindbis:Virus alone vs. mSindbis:bsIgG_1_**^E2^**^xHER2^ | 0.0156 | * |
| WT Sindbis:Virus alone vs. mSindbis:tandem Fab**^E2^**^xHER2^ | <0.0001 | **** |
| WT Sindbis:Virus alone vs. mSindbis:Virus + IgG_1_^HER2^ | 0.0082 | ** |
| WT Sindbis:bsIgG_1_**^E2^**^xHER2^ vs. WT Sindbis:tandem Fab**^E2^**^xHER2^ | <0.0001 | **** |
| WT Sindbis:bsIgG_1_**^E2^**^xHER2^ vs. WT Sindbis:Virus + IgG_1_^HER2^ | <0.0001 | **** |
| WT Sindbis:bsIgG_1_**^E2^**^xHER2^ vs. mSindbis:Virus alone | <0.0001 | **** |
| WT Sindbis:bsIgG_1_**^E2^**^xHER2^ vs. mSindbis:bsIgG_1_**^E2^**^xHER2^ | <0.0001 | **** |
| WT Sindbis:bsIgG_1_**^E2^**^xHER2^ vs. mSindbis:tandem Fab**^E2^**^xHER2^ | <0.0001 | **** |
| WT Sindbis:bsIgG_1_**^E2^**^xHER2^ vs. mSindbis:Virus + IgG_1_^HER2^ | <0.0001 | **** |
| WT Sindbis:tandem Fab**^E2^**^xHER2^ vs. WT Sindbis:Virus + IgG_1_^HER2^ | <0.0001 | **** |
| WT Sindbis:tandem Fab**^E2^**^xHER2^ vs. mSindbis:Virus alone | <0.0001 | **** |
| WT Sindbis:tandem Fab**^E2^**^xHER2^ vs. mSindbis:bsIgG_1_**^E2^**^xHER2^ | <0.0001 | **** |
| WT Sindbis:tandem Fab**^E2^**^xHER2^ vs. mSindbis:tandem Fab**^E2^**^xHER2^ | <0.0001 | **** |
| WT Sindbis:tandem Fab**^E2^**^xHER2^ vs. mSindbis:Virus + IgG_1_^HER2^ | <0.0001 | **** |
| WT Sindbis:Virus + IgG_1_^HER2^ vs. mSindbis:Virus alone | 0.0005 | *** |
| WT Sindbis:Virus + IgG_1_^HER2^ vs. mSindbis:bsIgG_1_**^E2^**^xHER2^ | 0.2846 | ns |
| WT Sindbis:Virus + IgG_1_^HER2^ vs. mSindbis:tandem Fab**^E2^**^xHER2^ | 0.0002 | *** |
| WT Sindbis:Virus + IgG_1_^HER2^ vs. mSindbis:Virus + IgG_1_^HER2^ | 0.0014 | ** |
| mSindbis:Virus alone vs. mSindbis:bsIgG_1_**^E2^**^xHER2^ | <0.0001 | **** |
| mSindbis:Virus alone vs. mSindbis:tandem Fab**^E2^**^xHER2^ | <0.0001 | **** |
| mSindbis:Virus alone vs. mSindbis:Virus + IgG_1_^HER2^ | >0.9999 | ns |
| mSindbis:bsIgG_1_**^E2^**^xHER2^ vs. mSindbis:tandem Fab**^E2^**^xHER2^ | 0.0877 | ns |
| mSindbis:bsIgG_1_**^E2^**^xHER2^ vs. mSindbis:Virus + IgG_1_^HER2^ | <0.0001 | **** |
| mSindbis:tandem Fab**^E2^**^xHER2^ vs. mSindbis:Virus + IgG_1_^HER2^ | <0.0001 | **** |
