# Supplementary material for: Efficient and Highly Specific Gene Transfer Using Mutated Lentiviral Vectors Redirected with Bispecific Antibodies
Source: mBio. 2020 Jan 21;11(1):e02990-19. doi: 10.1128/mBio.02990-19 (PMC6989108; doi:10.1128/mBio.02990-19)
Supplement: TABLE S8 [file mBio.02990-19-st008.docx]

**Table S8**

| **Treatment Comparisons for Fig. 5B** | **Adjusted P Value** | **Summary** |
| --- | --- | --- |
| WT Sindbis:Virus alone vs. WT Sindbis:Virus + bsIgG_1_**^E2^**^xHER2^ | <0.0001 | **** |
| WT Sindbis:Virus alone vs. WT Sindbis:Virus + tandem Fab**^E2^**^xHER2^ | <0.0001 | **** |
| WT Sindbis:Virus alone vs. WT Sindbis:Virus + IgG_1_^HER2^ | 0.9996 | ns |
| WT Sindbis:Virus alone vs. mSindbis:Virus alone | 0.5993 | ns |
| WT Sindbis:Virus alone vs. mSindbis:Virus + bsIgG_1_**^E2^**^xHER2^ | 0.453 | ns |
| WT Sindbis:Virus alone vs. mSindbis:Virus + tandem Fab**^E2^**^xHER2^ | 0.023 | * |
| WT Sindbis:Virus alone vs. mSindbis:Virus + IgG_1_^HER2^ | 0.7047 | ns |
| WT Sindbis:Virus + bsIgG_1_**^E2^**^xHER2^ vs. WT Sindbis:Virus + tandem Fab**^E2^**^xHER2^ | 0.0009 | *** |
| WT Sindbis:Virus + bsIgG_1_**^E2^**^xHER2^ vs. WT Sindbis:Virus + IgG_1_^HER2^ | <0.0001 | **** |
| WT Sindbis:Virus + bsIgG_1_**^E2^**^xHER2^ vs. mSindbis:Virus alone | <0.0001 | **** |
| WT Sindbis:Virus + bsIgG_1_**^E2^**^xHER2^ vs. mSindbis:Virus + bsIgG_1_**^E2^**^xHER2^ | <0.0001 | **** |
| WT Sindbis:Virus + bsIgG_1_**^E2^**^xHER2^ vs. mSindbis:Virus + tandem Fab**^E2^**^xHER2^ | <0.0001 | **** |
| WT Sindbis:Virus + bsIgG_1_**^E2^**^xHER2^ vs. mSindbis:Virus + IgG_1_^HER2^ | <0.0001 | **** |
| WT Sindbis:Virus + tandem Fab**^E2^**^xHER2^ vs. WT Sindbis:Virus + IgG_1_^HER2^ | <0.0001 | **** |
| WT Sindbis:Virus + tandem Fab**^E2^**^xHER2^ vs. mSindbis:Virus alone | <0.0001 | **** |
| WT Sindbis:Virus + tandem Fab**^E2^**^xHER2^ vs. mSindbis:Virus + bsIgG_1_**^E2^**^xHER2^ | <0.0001 | **** |
| WT Sindbis:Virus + tandem Fab**^E2^**^xHER2^ vs. mSindbis:Virus + tandem Fab**^E2^**^xHER2^ | <0.0001 | **** |
| WT Sindbis:Virus + tandem Fab**^E2^**^xHER2^ vs. mSindbis:Virus + IgG_1_^HER2^ | <0.0001 | **** |
| WT Sindbis:Virus + IgG_1_^HER2^ vs. mSindbis:Virus alone | 0.305 | ns |
| WT Sindbis:Virus + IgG_1_^HER2^ vs. mSindbis:Virus + bsIgG_1_**^E2^**^xHER2^ | 0.7322 | ns |
| WT Sindbis:Virus + IgG_1_^HER2^ vs. mSindbis:Virus + tandem Fab**^E2^**^xHER2^ | 0.0657 | ns |
| WT Sindbis:Virus + IgG_1_^HER2^ vs. mSindbis:Virus + IgG_1_^HER2^ | 0.4255 | ns |
| mSindbis:Virus alone vs. mSindbis:Virus + bsIgG_1_**^E2^**^xHER2^ | 0.0163 | * |
| mSindbis:Virus alone vs. mSindbis:Virus + tandem Fab**^E2^**^xHER2^ | 0.0003 | ******* |
| mSindbis:Virus alone vs. mSindbis:Virus + IgG_1_^HER2^ | >0.9999 | ns |
| mSindbis:Virus + bsIgG_1_**^E2^**^xHER2^ vs. mSindbis:Virus + tandem Fab**^E2^**^xHER2^ | 0.8607 | ns |
| mSindbis:Virus + bsIgG_1_**^E2^**^xHER2^ vs. mSindbis:Virus + IgG_1_^HER2^ | 0.034 | * |
| mSindbis:Virus + tandem Fab**^E2^**^xHER2^ vs. mSindbis:Virus + IgG_1_^HER2^ | 0.001 | *** |
| **Treatment Comparisons for Fig. 5C** | **Adjusted P Value** | **Summary** |
| WT Sindbis:tandem Fab vs. WT Sindbis:tandem Fab + 0.1 μM αHER2 IgG1 | <0.0001 | **** |
| WT Sindbis:tandem Fab vs. mSindbis:tandem Fab | <0.0001 | **** |
| WT Sindbis:tandem Fab vs. mSindbis:tandem Fab + 0.1 μM αHER2 IgG1 | <0.0001 | **** |
| WT Sindbis:Group B vs. mSindbis:tandem Fab | 0.0005 | *** |
| WT Sindbis:Group B vs. mSindbis:tandem Fab + 0.1 μM αHER2 IgG1 | 0.1282 | ns |
| mSindbis:tandem Fab vs. mSindbis:tandem Fab + 0.1 μM αHER2 IgG1 | 0.0002 | *** |
