# Supplementary material for: Efficient and Highly Specific Gene Transfer Using Mutated Lentiviral Vectors Redirected with Bispecific Antibodies
Source: mBio. 2020 Jan 21;11(1):e02990-19. doi: 10.1128/mBio.02990-19 (PMC6989108; doi:10.1128/mBio.02990-19)
Supplement: TABLE S9 [file mBio.02990-19-st009.docx]

**Table S9**

| **Treatment Comparisons** | **Adjusted P Value** | **Summary** |
| --- | --- | --- |
| WT Sindbis:Virus alone vs. WT Sindbis:Virus + bsIgG_1_**^E1xHER2^** | 0.1192 | ns |
| WT Sindbis:Virus alone vs. WT Sindbis:Virus + bsIgG_1_**^E2xHER2^** | 0.9911 | ns |
| WT Sindbis:Virus alone vs. WT Sindbis:Virus + tandem Fab**^E2xHER2^** | 0.0301 | * |
| WT Sindbis:Virus alone vs. WT Sindbis:Virus + IgG_1_^HER2^ | 0.1157 | ns |
| WT Sindbis:Virus alone vs. mSindbis:Virus alone | 0.9984 | ns |
| WT Sindbis:Virus alone vs. mSindbis:Virus + bsIgG_1_**^E1xHER2^** | 0.0019 | ** |
| WT Sindbis:Virus alone vs. mSindbis:Virus + bsIgG_1_**^E2xHER2^** | 0.9773 | ns |
| WT Sindbis:Virus alone vs. mSindbis:Virus + tandem Fab**^E2xHER2^** | 0.0024 | ** |
| WT Sindbis:Virus alone vs. mSindbis:Virus + IgG_1_^HER2^ | 0.0515 | ns |
| WT Sindbis:Virus + bsIgG_1_**^E1xHER2^** vs. WT Sindbis:Virus + bsIgG_1_**^E2xHER2^** | 0.6546 | ns |
| WT Sindbis:Virus + bsIgG_1_**^E1xHER2^** vs. WT Sindbis:Virus + tandem Fab**^E2xHER2^** | 0.999 | ns |
| WT Sindbis:Virus + bsIgG_1_**^E1xHER2^** vs. WT Sindbis:Virus + IgG_1_^HER2^ | >0.9999 | ns |
| WT Sindbis:Virus + bsIgG_1_**^E1xHER2^** vs. mSindbis:Virus alone | 0.5131 | ns |
| WT Sindbis:Virus + bsIgG_1_**^E1xHER2^** vs. mSindbis:Virus + bsIgG_1_**^E1xHER2^** | 0.9186 | ns |
| WT Sindbis:Virus + bsIgG_1_**^E1xHER2^** vs. mSindbis:Virus + bsIgG_1_**^E2xHER2^** | 0.747 | ns |
| WT Sindbis:Virus + bsIgG_1_**^E1xHER2^** vs. mSindbis:Virus + tandem Fab**^E2xHER2^** | 0.8582 | ns |
| WT Sindbis:Virus + bsIgG_1_**^E1xHER2^** vs. mSindbis:Virus + IgG_1_^HER2^ | >0.9999 | ns |
| WT Sindbis:Virus + bsIgG_1_**^E2xHER2^** vs. WT Sindbis:Virus + tandem Fab**^E2xHER2^** | 0.272 | ns |
| WT Sindbis:Virus + bsIgG_1_**^E2xHER2^** vs. WT Sindbis:Virus + IgG_1_^HER2^ | 0.646 | ns |
| WT Sindbis:Virus + bsIgG_1_**^E2xHER2^** vs. mSindbis:Virus alone | >0.9999 | ns |
| WT Sindbis:Virus + bsIgG_1_**^E2xHER2^** vs. mSindbis:Virus + bsIgG_1_**^E1xHER2^** | 0.0424 | * |
| WT Sindbis:Virus + bsIgG_1_**^E2xHER2^** vs. mSindbis:Virus + bsIgG_1_**^E2xHER2^** | >0.9999 | ns |
| WT Sindbis:Virus + bsIgG_1_**^E2xHER2^** vs. mSindbis:Virus + tandem Fab**^E2xHER2^** | 0.041 | * |
| WT Sindbis:Virus + bsIgG_1_**^E2xHER2^** vs. mSindbis:Virus + IgG_1_^HER2^ | 0.4287 | ns |
| WT Sindbis:Virus + tandem Fab**^E2xHER2^** vs. WT Sindbis:Virus + IgG_1_^HER2^ | 0.9991 | ns |
| WT Sindbis:Virus + tandem Fab**^E2xHER2^** vs. mSindbis:Virus alone | 0.1856 | ns |
| WT Sindbis:Virus + tandem Fab**^E2xHER2^** vs. mSindbis:Virus + bsIgG_1_**^E1xHER2^** | >0.9999 | ns |
| WT Sindbis:Virus + tandem Fab**^E2xHER2^** vs. mSindbis:Virus + bsIgG_1_**^E2xHER2^** | 0.345 | ns |
| WT Sindbis:Virus + tandem Fab**^E2xHER2^** vs. mSindbis:Virus + tandem Fab**^E2xHER2^** | 0.9989 | ns |
| WT Sindbis:Virus + tandem Fab**^E2xHER2^** vs. mSindbis:Virus + IgG_1_^HER2^ | >0.9999 | ns |
| WT Sindbis:Virus + IgG_1_^HER2^ vs. mSindbis:Virus alone | 0.5045 | ns |
| WT Sindbis:Virus + IgG_1_^HER2^ vs. mSindbis:Virus + bsIgG_1_**^E1xHER2^** | 0.9227 | ns |
| WT Sindbis:Virus + IgG_1_^HER2^ vs. mSindbis:Virus + bsIgG_1_**^E2xHER2^** | 0.7392 | ns |
| WT Sindbis:Virus + IgG_1_^HER2^ vs. mSindbis:Virus + tandem Fab**^E2xHER2^** | 0.8635 | ns |
| WT Sindbis:Virus + IgG_1_^HER2^ vs. mSindbis:Virus + IgG_1_^HER2^ | >0.9999 | ns |
| mSindbis:Virus alone vs. mSindbis:Virus + bsIgG_1_**^E1xHER2^** | 0.0236 | * |
| mSindbis:Virus alone vs. mSindbis:Virus + bsIgG_1_**^E2xHER2^** | >0.9999 | ns |
| mSindbis:Virus alone vs. mSindbis:Virus + tandem Fab**^E2xHER2^** | 0.0238 | * |
| mSindbis:Virus alone vs. mSindbis:Virus + IgG_1_^HER2^ | 0.3044 | ns |
| mSindbis:Virus + bsIgG_1_**^E1xHER2^** vs. mSindbis:Virus + bsIgG_1_**^E2xHER2^** | 0.0623 | ns |
| mSindbis:Virus + bsIgG_1_**^E1xHER2^** vs. mSindbis:Virus + tandem Fab**^E2xHER2^** | >0.9999 | ns |
| mSindbis:Virus + bsIgG_1_**^E1xHER2^** vs. mSindbis:Virus + IgG_1_^HER2^ | 0.9854 | ns |
| mSindbis:Virus + bsIgG_1_**^E2xHER2^** vs. mSindbis:Virus + tandem Fab**^E2xHER2^** | 0.0587 | ns |
| mSindbis:Virus + bsIgG_1_**^E2xHER2^** vs. mSindbis:Virus + IgG_1_^HER2^ | 0.5246 | ns |
| mSindbis:Virus + tandem Fab**^E2xHER2^** vs. mSindbis:Virus + IgG_1_^HER2^ | 0.9597 | ns |
